# Caffeine, but not paracetamol (acetaminophen), enhances muscular endurance, strength, and power

**DOI:** 10.1080/15502783.2024.2400513

**Published:** 2024-09-08

**Authors:** Bela Scapec, Jozo Grgic, Dorian Varovic, Pavle Mikulic

**Affiliations:** a University of Zagreb Faculty of Kinesiology, Zagreb, Croatia; b National University of Singapore, Healthy Longevity Translational Research Program, Yong Loo Lin School of Medicine, Singapore; c National University Health System, Centre for Healthy Longevity, Singapore

**Keywords:** Analgesics, ergogenic aids, interaction, supplements

## Abstract

**Background:**

Caffeine is one of the most popular ergogenic aids consumed by athletes. Caffeine’s ergogenic effect has been generally explained by its ability to bind to adenosine receptors, thus modulating pain and reducing perceived exertion. Another pharmacological agent that may improve performance due to its analgesic proprieties is paracetamol. This study aimed to explore the effects of caffeine, paracetamol, and caffeine + paracetamol consumption on muscular endurance, strength, power, anaerobic endurance, and jumping performance.

**Methods:**

In this randomized, crossover, double-blind study, 29 resistance-trained participants (11 men and 18 women) ingested either a placebo, caffeine (3 mg/kg), paracetamol (1500 mg) or caffeine + paracetamol 45 min before the testing sessions. The testing sessions included performing the bench press exercise with 75% of one-repetition maximum to momentary muscular failure, isokinetic knee extension and flexion at angular velocities of 60°/sec and 180°/sec, Wingate, and countermovement jump (CMJ) tests.

**Results:**

Compared to placebo, isolated caffeine ingestion increased the number of repetitions performed in the bench press (*p* = 0.005; *d* = 0.42). Compared to placebo, isolated caffeine ingestion and/or caffeine + paracetamol consumption was ergogenic for strength (torque), muscular endurance (total work), or power in the isokinetic assessment, particularly at slower angular velocities (*p* = 0.027 to 0.002; *d* = 0.16 to 0.26). No significant differences between the conditions were observed for outcomes related to the Wingate and CMJ tests.

**Conclusion:**

This study provided novel evidence into the effectiveness of caffeine, paracetamol, and their combination on exercise performance. We found improvements in muscular endurance, strength, or power only when caffeine was consumed in isolation, or in combination with paracetamol. Isolated paracetamol consumption did not improve performance for any of the analyzed outcomes, thus calling into question its ergogenic potential.

## 1. Introduction

Caffeine is one of the most popular ergogenic aids, commonly consumed by athletes and non-athletes alike [1]. Data indicates that caffeine ingestion may acutely enhance various components of exercise performance, such as power, muscular strength, muscular and aerobic endurance, and jumping performance [2]. Caffeine is well-researched and has been identified by the International Olympic Committee as one of the supplements for which there is good evidence supporting its ergogenic effect [3]. Still, while the effects of caffeine – and many other supplements – have been explored in isolation, less is generally known about supplement interactions [4]. Many athletes often ingest more than one dietary supplement at once, and multi-ingredient blends (e.g. pre-workout drinks) are commercially available, which may contain up to 30 individual substances [4,5]. While a given supplement may be ergogenic when provided in isolation, a combination with another substance may moderate these effects [4]. Therefore, there is a growing need to explore the effects of combining different substances on exercise performance.

While the exact mechanisms underpinning the performance improvements are not yet fully clear, caffeine’s ergogenic effect has been generally explained by its ability to bind to adenosine receptors, thus modulating pain and reducing perceived exertion [6]. Another pharmacological agent that may improve performance due to its analgesic proprieties is paracetamol (acetaminophen) [7]. Paracetamol is commonly used for fever reduction and pain relief [8]. Paracetamol’s pain-decreasing effects are related to the inhibition of prostaglandin synthesis [9]. This inhibition reduces the transduction of the sensory nerves and decreases nociceptive impulse transmission, likely explaining paracetamol’s effects on pain modulation [7,9,10]. Due to these effects and the reports of paracetamol use among athletes, researchers have also explored the effects of paracetamol on exercise performance [7,11–23]. For example, one study [14] examined the effects of consuming 1500 mg of paracetamol 60 minutes before performing a cycling time trial (16.1 km). Compared to placebo, paracetamol consumption reduced the time needed to complete the task by 30 seconds [14]. Follow-up research by the same group also established an ergogenic effect of paracetamol on endurance performance in the heat (30°C), where paracetamol consumption improved performance in a task involving cycling to exhaustion [15].

The preponderance of studies exploring paracetamol’s effects on exercise performance focused on aerobic endurance [14–19]. Thus, there is a scarcity of studies investigating the effects of this pharmacological agent on other components of exercise performance [7]. A recent review summarized the currently available evidence and reported there are only preliminary data indicating that paracetamol ingestion may be ergogenic for muscular strength and sprint performance [7,20–23]. Specifically, studies have reported that paracetamol ingestion may enhance: (a) peak and mean power in the Wingate test; and (b) mean and critical torque [20,22,23]. For outcomes such as muscular endurance paracetamol’s effects remain unclear, due to the paucity of data [7,20–23]. Even though caffeine is commonly combined with analgesics (e.g. paracetamol) due to their additive effects for pain relief [24], only two studies [16,17] explored their combined effects on exercise performance, and both only focused on cycling time trials, highlighting another gap in the research.

The aim of the present study was to explore the isolated and combined effects of caffeine and paracetamol on muscular endurance, strength, power, anaerobic endurance, and jumping performance. We hypothesized that isolated consumption of caffeine and paracetamol would improve performance and that their combination would produce the greatest effect.

## 2. Materials and methods

### 2.1. Experimental design

**Figure 1.**
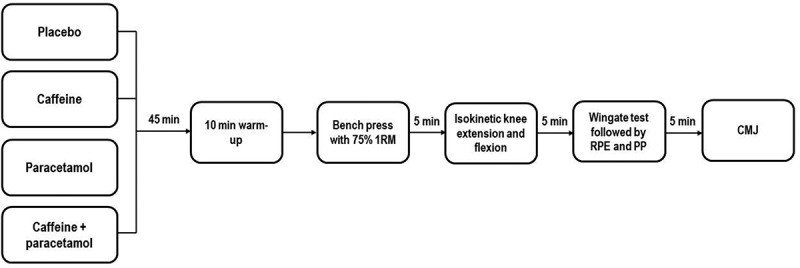
Depiction of the experimental protocol (order of the conditions was randomized). Minutes denote rest interval time between the tests. 1RM: one repetition maximum, RPE: completing the rating of perceived exertion scale; PP: completing the pain perception scale.

This study utilized a randomized, crossover, double-blind design. The participants completed five visits to the laboratory. In the first visit, the participants performed a one-repetition maximum (1RM) testing in the bench press and familiarization with the testing protocol. After the familiarization visit, the participants completed four main testing conditions, which included the consumption of: caffeine, paracetamol, caffeine + paracetamol, or placebo. The main testing sessions were completed in a randomized and counterbalanced manner, four to seven days apart. In the main testing session, the participants completed the bench press exercise with 75% of 1RM to momentary muscular failure, isokinetic knee extension and flexion, a Wingate test, and a countermovement jump test (CMJ) – performed in that order (Figure 1). These tests have been selected to provide a comprehensive overview of the effectiveness of caffeine and paracetamol on exercise performance. The bench press exercise is commonly used in resistance exercise programs and mainly activates the pectoralis, deltoid, and triceps muscles. We opted to use 75% of 1RM as this load is commonly prescribed when training for outcomes such as hypertrophy and strength [25]. Isokinetic testing was also employed due to its benefits such as: (a) providing maximal resistance throughout the range of motion; (b) use of accommodating resistance; and (c) providing outputs such as torque, work, and power [26]. Wingate testing was utilized to examine the effects of caffeine and paracetamol in an exercise where energy contributions vary (i.e. ~16% aerobic, ~56% glycolytic, ~28% adenosine triphosphate and phosphocreatine) and because its outcomes (i.e. mean and peak power) correlate with performance outcomes in sports cycling, speed skating, and ice hockey [27–30]. Finally, CMJ testing was also used as jumping tasks are ubiquitous in many sports and to evaluate the effects of caffeine and paracetamol on performance in exercise tasks where phosphocreatine and adenosine triphosphate are the primary energy sources. A five-minute rest interval was provided between each test, which was estimated to be sufficient for complete or near-complete recovery. All testing was performed in the morning hours (7–9 AM). In the 24 hours before visiting the laboratory, the participants were required not to perform any strenuous exercise. The participants fasted overnight and only consumed one banana prior to visiting the laboratory and ingesting the capsules. This light meal/snack was provided to reduce the likelihood of nausea related to supplementation and to follow the current guidelines suggesting not to exercise in a fasted state [31]. The caffeine, paracetamol, and placebo (maltodextrin) powders were weighed using a high-precision electronic digital scale and provided in gelatin capsules of identical appearance to maintain a double-blind design. Capsules contained either caffeine (3 mg/kg), paracetamol (1500 mg) or placebo (1000 mg maltodextrin). In each trial, capsules were ingested 45 minutes before the testing started. The number of consumed capsules was the same in each trial (i.e. caffeine + placebo; paracetamol + placebo; placebo + placebo; caffeine + paracetamol) to ensure the maintenance of a double-blind study design.

### 2.2. Participants

Prospective participants were considered eligible for this study if they satisfied the following criteria: (a) 18–45 years old; (b) minimum 1 year of resistance training experience; and (c) possessing the ability to lift 100% and 60% of their body mass in the bench press exercise for men and women, respectively. Exclusion criteria included the existence of any health limitations, contraindications pertaining to caffeine and/or paracetamol ingestion (assessed via a questionnaire), and prior use of anabolic steroids (self-reported). We initially recruited a sample of 35 resistance-trained men and women. Six participants did not complete all testing sessions (five due to a loss of interest/personal reasons and one sustained an injury not related to the study). Thus, a total of 29 participants (11 men and 18 women) completed all testing sessions (age: 25 ± 4.0 years; height: 172 ± 8.5 cm; body mass: 70 ± 13 kg; 1RM [all]: 70 ± 29 kg; 1RM [women]: 50 ± 9 kg; 1RM [men]: 100 ± 20 kg). Before enrolling in the study, every participant signed an informed consent form. The Committee for Scientific Research and Ethics of the University of Zagreb Faculty of Kinesiology provided ethical approval for the study (approval number 27/2023; document dated 3 April 2023).

### 2.3. 1RM testing

Participants performed a self-selected 10-minute warm-up before commencing the 1RM testing. At the beginning of the protocol, the participants performed 4 warm-up sets using an empty Olympic barbell (20 kg), 50, 75, and 95% of their estimated 1RM for 8–10, 8–10, 3–6, and 1 repetition, respectively. After the warm-up, 1RM attempts were performed, with progressive increases in load each subsequent attempt until a true 1RM value was obtained. 1RMs were determined within five attempts for all participants. A successful 1RM attempt was deemed when a participant was able to complete the concentric portion of the repetition without any assistance. Three-minute rest intervals were provided between attempts. Following the completion of 1RM testing, participants were familiarized with the main testing protocol (Figure 1).

### 2.4. Bench press

All testing sessions started with the bench press exercise, preceded by a self-selected 10-minute warm-up. We instructed participants to maintain the same warm-up routine in all testing sessions. A specific warm-up for the bench press testing was performed using an empty Olympic barbell (20 kg), followed by 50% of participants 1RM for 8–10 repetitions. The exercise was performed in a supine position on a flat bench. We instructed the participants to maintain five points of contact (head, shoulder blades, and glutes in contact with the bench and both feet in contact with the ground) at all times when performing the exercise. In the eccentric part of the movement, the barbell was required to be lowered to the middle or lower portion of the pectoralis muscle. The end of the movement (i.e. completion of a given repetition) was the moment when the participants lifted the barbell and achieved a full extension of their elbows. Participants were instructed to perform the concentric portion of the lift with maximal effort and velocity, while the eccentric portion lasted 1–2 seconds (no pause at the bottom). The bench press exercise was performed to the point of momentary muscular failure using 75% of 1RM.

### 2.5. Isokinetic testing

Using angular velocities of 60°/sec and 180°/sec (tested in that order), we evaluated peak torque, total work, and average power of the knee extensors and knee flexors on an isokinetic dynamometer (System 4 Pro, Biodex Medical Systems, Inc., Shirley, NY, USA). These assessments were only performed for the dominant leg. Before each testing session, the dynamometer was calibrated. After placing the participants in a seated position on the testing device, we applied stabilization straps to the shin, thigh, waist, and trunk. The participants were positioned in a manner that the lateral femoral epicondyle of the leg performing the extension/flexion is aligned with the dynamometer’s axis of rotation. The range of motion of the knee joint in this test was set at 80°. For both angular velocities, the participants initially performed three familiarization repetitions to get accustomed to the speed of the lever arm. After the familiarization repetitions, a 30-second rest interval was provided. Then, five maximal knee extensions and flexions were performed, with instructions to extend and flex the knee (to “kick” and “pull”) while giving maximum effort.

### 2.6. Wingate test

The Wingate test was performed on a bicycle ergometer (Ergomedic 894E, Monark, Sweden). Saddle height was set individually during the first visit and used for all subsequent visits. The test started with a standardized warm-up protocol that consisted of unloaded pedaling for 3 minutes at 60–80 rpm [32]. In the last 3 seconds of each minute, participants performed a maximum-intensity sprint. After completing the warm-up, participants continued to sit on the bicycle and passively rested for 1 minute. Then, participants were asked to pedal at their maximum pedaling speed, after which the resistance (7.5% of the participant’s body weight) was applied, and participants performed a 30-second “all-out” sprint. We instructed the participants to pedal at their maximum speed while remaining seated for the duration of the test. Upon completion of the test, the participants performed a 2-minute unloaded cool-down pedaling at 60–80 rpm. Peak power, mean power, minimum power, and power drop were recorded using the Monark anaerobic test software (version 3.3.0.0.).

### 2.7. CMJ

The participants performed three CMJs on a force platform (BP600600, AMTI, Inc., Watertown, MA, USA), which has software for data acquisition and analysis. The participants started from an upright standing position. Following the testers’ command, the participants were required to perform a fast knee flexion (i.e. a downward countermovement), where their lowest point should be a semi-squat position (knee ~90° and trunk/hips in a flexed position), immediately followed by a fast extension of the legs. Before each testing session, we reminded the participants that the goal is to jump as “explosively” and quickly as possible, to reach maximum jump height. Three official attempts were performed following one warm-up attempt (1 min rest between attempts). Analyzed performance outcomes included vertical jump height (automatically calculated by the software from flight time), maximum power, contraction time, flight time, and flight time to contraction time ratio.

### 2.8. Rating of perceived exertion (RPE) and pain perception (PP)

Following the completion of the Wingate test, participants were asked to indicate their perceived levels of exertion on the RPE scale [33], as well as their perceived levels of pain on a previously validated scale [34]. For the RPE scale, the responses ranged from 6 to 20, while on the pain perception scale, the responses ranged from 0 to 10.

### 2.9. Assessment of blinding

To evaluate how effective was our double-blind design, we used an assessment procedure proposed by Saunders and colleagues [35]. This procedure was performed at two-time points, before and after the testing sessions. In both cases, we asked the participants to respond to the following question: “Please state which supplement you think you have ingested?.” The participants were able to choose one of five answers: (a) “paracetamol”; (b) “caffeine”; (c) “paracetamol + caffeine”; (d) “placebo; and (e) “I do not know.” If the participants did not select (e), they were required to state the reason for choosing their respective response.

### 2.10. Assessment of side effects

The incidence of side effects associated with the treatments provided was evaluated at two time points: (a) immediately after the completion of the testing sessions; and (b) in the following mornings, upon waking. The participants completed a survey, which consisted of 19 items regarding some of the most common side effects of caffeine and paracetamol.

### 2.11. Statistics

The data on exercise performance and perceptual responses (RPE and PP) were analyzed using repeated measures analysis of variance (ANOVA). When there was a significant main effect, we performed post hoc comparisons using Dunnett’s test, where the placebo condition was compared with the three other conditions (i.e. placebo vs. caffeine, placebo vs. paracetamol, and placebo vs. caffeine + paracetamol). The statistical significance threshold was set at *p* < 0.05. Relative effect sizes were calculated using Cohen’s *d* with 95% confidence intervals (95% CI) for repeated measures. Effect sizes of < 0.20, 0.20 to 0.49, 0.50 to 0.79, and ≥ 0.80 were considered to represent trivial, small, moderate, and large effects, respectively. The McNemar test was used to explore the differences in the incidence of side effects between the conditions. All analyses were performed using the STATISTICA software (version 14.1.0.8; TIBCO Software Inc. Paolo Alto, CA, USA).

## 3. Results

### 3.1. Bench press

**Figure 2.**
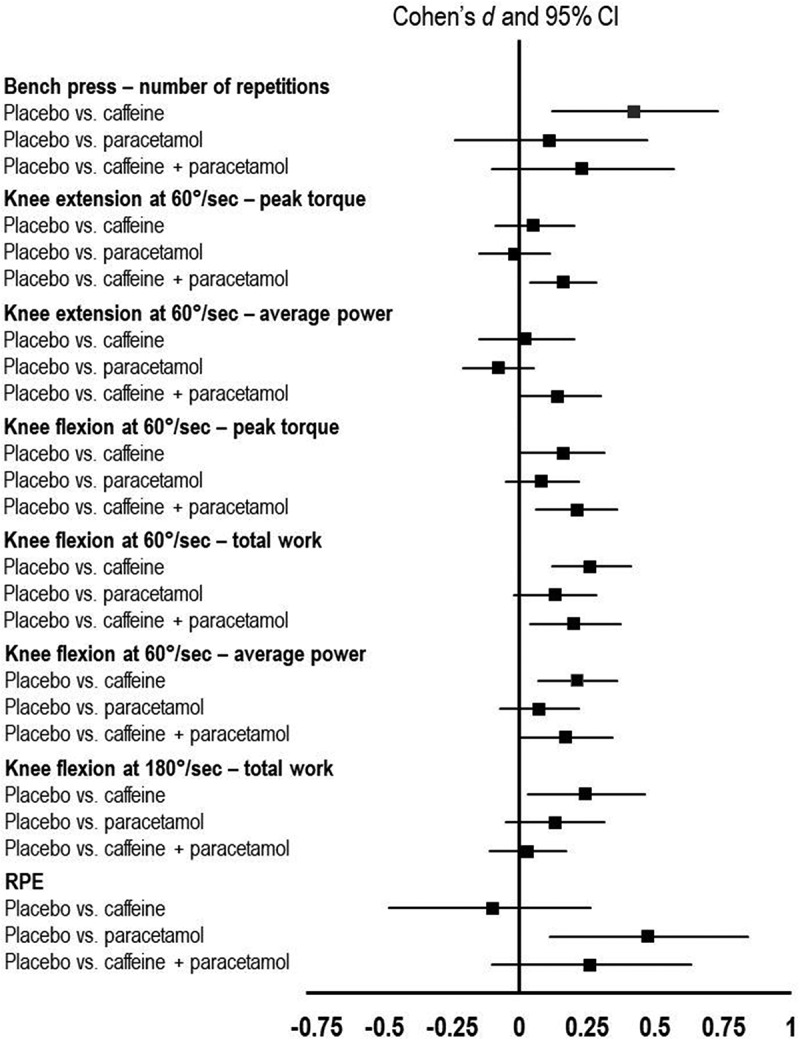
Summary of the study comparison between the conditions. Note: pairwise comparisons are made only for the outcome where a significant main effect was found in the repeated measures ANOVA; CI: confidence interval; RPE: rating of perceived exertion.

For the number of repetitions in the bench press, there was a significant main effect of condition (*p* = 0.028; Table 1). The post hoc test revealed that caffeine increased the number of repetitions performed (*p* = 0.005; *d* = 0.42; Figure 2). No significant differences were observed for paracetamol (*p* = 0.417) and caffeine + paracetamol (*p* = 0.122).

**Table 1.** Summary of the exercise performance data and the responses to the rating of perceived exertion and pain perception scales under the four employed conditions.

Test	Variable	Placebo	Caffeine	Paracetamol	Caffeine and paracetamol
Barbell bench press*	Number of repetitions	13.1 ± 2.8	14.3 ± 2.8	13.4 ± 2.3	13.8 ± 3.1
Isokinetic knee extension at 60°/sec	Peak torque (Nm)	168.5 ± 44.9	171.0 ± 46.1	167.6 ± 42.5	176.0 ± 47.7
	Total work (J)	460.0 ± 123.7	467.7 ± 126.3	457.7 ± 113.4	480.5 ± 136.1
	Average power (W)	108.1 ± 31.7	108.8 ± 30.9	105.7 ± 27.2	112.9 ± 33.6
Isokinetic knee flexion at 60°/sec	Peak torque (Nm)	92.5 ± 22.6	96.1 ± 22.9	94.6 ± 26.0	97.7 ± 26.4
	Total work (J)	307.3 ± 77.7	328.0 ± 77.1	317.8 ± 84.1	324.5 ± 89.5
	Average power (W)	68.9 ± 19.5	73.0 ± 18.4	70.3 ± 19.4	72.6 ± 22.4
Isokinetic knee extension at 180°/sec	Peak torque (Nm)	121.4 ± 35.6	122.9 ± 34.1	123.9 ± 31.3	123.0 ± 37.5
	Total work (J)	587.4 ± 178.5	601.7 ± 176.7	599.6 ± 150.1	594.6 ± 187.9
	Average power (W)	199.6 ± 67.8	203.7 ± 61.0	202.2 ± 52.0	200.7 ± 69.4
Isokinetic knee flexion at 180°/sec	Peak torque (Nm)	78.4 ± 19.5	82.4 ± 17.9	81.4 ± 19.9	79.4 ± 20.6
	Total work (J)	362.5 ± 122.9	391.0 ± 107.4	377.5 ± 107.0	366.5 ± 131.4
	Average power (W)	113.4 ± 41.2	121.1 ± 35.2	116.6 ± 37.2	113.3 ± 41.9
Wingate	Peak power (W)	585.4 ± 164.2	601.4 ± 183.1	597.7 ± 179.8	591.0 ± 163.6
	Mean power (W)	438.5 ± 115.3	447.1 ± 126.3	442.2 ± 123.4	444.6 ± 119.0
	Minimum power (W)	283.4 ± 75.4	283.9 ± 85.1	287.8 ± 81.0	275.4 ± 87.8
	Power drop (%)	50.8 ± 8.4	51.5 ± 13.0	50.9 ± 9.3	52.9 ± 10.9
RPE after the Wingate test	Arbitrary units (6-20 scale)	15.6 ± 2.9	15.4 ± 2.7	16.8 ± 2.0	16.3 ± 2.3
PP after the Wingate test	Arbitrary units (0-10 scale)	4.1 ± 2.4	3.5 ± 2.4	4.1 ± 2.4	3.7 ± 2.4
CMJ	Jump height (cm)	27.4 ± 5.8	27.3 ± 5.8	26.9 ± 5.3	27.6 ± 5.5
	Maximum power (W)	69.1 ± 10.4	68.3 ± 11.1	68.0 ± 8.6	69.8 ± 9.4
	Contraction time (s)	0.761 ± 0.084	0.813 ± 0.299	0.750 ± 0.092	0.745 ± 0.069
	Flight time (s)	0.470 ± 0.049	0.470 ± 0.050	0.467 ± 0.046	0.473 ± 0.047
	Flight time to contraction time	0.626 ± 0.100	0.612 ± 0.120	0.630 ± 0.091	0.640 ± 0.086

Note: RPE: rating of perceived exertion; PP: pain perception; CMJ: countermovement jump test; * data for the bench press were obtained from 28 participants.

### 3.2. Isokinetic knee extension at 60°/sec

For peak torque, there was a significant main effect of condition (*p* = 0.034). The post hoc test revealed that caffeine + paracetamol increased peak torque (*p* = 0.022; *d* = 0.16). No significant differences were observed for caffeine (*p* = 0.401) and paracetamol (*p* = 0.843).

For total work, there was no significant main effect (*p* = 0.055), and no post hoc analysis was performed.

For average power, there was a significant main effect of condition (*p* = 0.028). In the post hoc test, no significant differences were observed for caffeine (*p* = 0.626), paracetamol (*p* = 0.964) and caffeine + paracetamol (*p* = 0.055).

### 3.3. Isokinetic knee flexion at 60°/sec

For peak torque, there was a significant main effect of condition (*p* = 0.033). The post hoc test revealed that caffeine + paracetamol increased peak torque (*p* = 0.006; *d* = 0.21). No significant differences were observed for caffeine (*p* = 0.059) and paracetamol (*p* = 0.255).

For total work, there was a significant main effect (*p* = 0.009). The post hoc test revealed that caffeine (*p* = 0.002; *d* = 0.26) and caffeine + paracetamol (*p* = 0.011; *d* = 0.20) increased total work. No significant differences were observed for paracetamol (*p* = 0.119).

For average power, there was a significant main effect (*p* = 0.032). The post hoc test revealed that caffeine (*p* = 0.014; *d* = 0.21) and caffeine + paracetamol (*p* = 0.027; *d* = 0.17) increased average power. No significant differences were observed for paracetamol (*p* = 0.363).

### 3.4. Isokinetic knee extension at 180°/sec

There was no significant main effect for peak torque (*p* = 0.866), total work (*p* = 0.799), and average power (*p* = 0.891), and no post hoc analysis was performed.

### 3.5. Isokinetic knee flexion at 180°/sec

For peak torque, there was no significant main effect (*p* = 0.098), and no post hoc analysis was performed.

For total work, there was a significant main effect of condition (*p* = 0.038). The post hoc test revealed that caffeine increased total work (*p* = 0.011; *d* = 0.24). No significant differences were observed for paracetamol (*p* = 0.176) and caffeine + paracetamol (*p* = 0.597).

For average power, there was no significant main effect (*p* = 0.148), and no post hoc analysis was performed.

### 3.6. Wingate

There was no significant main effect for peak power (*p* = 0.465), mean power (*p* = 0.768), minimum power (*p* = 0.777), or power drop (*p* = 0.744), and no post hoc analysis was performed.

### 3.7. CMJ

There was no significant main effect for jump height (*p* = 0.182), maximum power (*p* = 0.438), contraction time (*p* = 0.256), flight time (*p* = 0.194), or flight time to contraction time (*p* = 0.326), and no post hoc analysis was performed.

### 3.8. RPE and PP

For RPE recorded following the Wingate test, there was a significant main effect of condition (*p* = 0.011). The post hoc test revealed that paracetamol consumption increased RPE (*p* = 0.046; *d* = 0.38). No significant differences were observed for caffeine (*p* = 0.823) and caffeine + paracetamol (*p* = 0.362).

For PP recorded following the Wingate test, there was no significant main effect (*p* = 0.483), and no post hoc analysis was performed.

### 3.9. Side effects

The incidence of side effects is presented in Table 2. There was no significant difference between the conditions (*p* > 0.05 for all).

**Table 2.** Incidence of side effects following the consumption of placebo, caffeine, paracetamol, and caffeine + paracetamol, evaluated after the testing session and in the following morning.

Side effect	After the testing session				Morning after the testing session			
	Placebo	Caffeine	Paracetamol	Caffeine + paracetamol	Placebo	Caffeine	Paracetamol	Caffeine + paracetamol
Rash	0	0	0	0	0	0	0	0
Itching	0	0	0	0	0	0	0	0
Swelling of the lips and face	0	1	0	0	0	0	0	0
Lack of breath	1	2	0	1	0	0	0	0
Skin peeling	0	0	0	0	0	0	0	0
Mouth ulcers	0	0	0	0	0	0	0	0
Brusing	0	0	0	0	0	0	0	0
Nausea	1	5	4	3	0	0	0	0
Lack of appetite	2	2	1	2	0	0	1	0
Yellow colored sclera and skin	0	0	0	0	0	0	0	0
Muscle soreness	1	1	2	2	0	0	2	0
Increased urine production	0	0	0	1	1	0	0	0
Tachycardia and heart palpitations	2	6	1	4	0	0	0	0
Increased anxiety	0	0	0	1	0	0	0	1
Headache	0	0	0	1	1	0	0	1
Abdominal/gut discomfort	0	1	0	1	0	0	0	0
Insomnia	n/a	n/a	n/a	n/a	0	0	0	0
Increased vigor/activeness	6	9	5	9	0	3	0	1
Perception of improved performance	10	10	6	13	n/a	n/a	n/a	n/a

**Note**: Data are reported as frequencies (number of positive cases) for the 29 included participants.

### 3.10 Blinding

In the pre-exercise assessment, the caffeine, placebo, paracetamol, and caffeine + paracetamol conditions were correctly identified by 2/29 (7%), 3/29 (10%), 3/29 (10%), and 3/29 (10%) of the participants, respectively. None of the participants correctly identified all the conditions.

In the post-exercise assessment, the caffeine, placebo, paracetamol, and caffeine + paracetamol conditions were correctly identified by 7/29 (24%), 5/29 (17%), 8/29 (28%), and 5/29 (17%) of the participants, respectively. None of the participants correctly identified all the conditions.

## 4. Discussion

We found that only isolated caffeine ingestion improved muscular endurance in the bench press. Isolated caffeine and/or caffeine + paracetamol ingestion was ergogenic for strength (torque), muscular endurance (total work), or power in the isokinetic assessment, particularly at slower angular velocities. We did not find an ergogenic effect on outcomes related to the Wingate and CMJ tests. In summary, improvements in performance were only observed when caffeine was consumed in isolation, or in combination with paracetamol. Isolated paracetamol consumption did not improve performance for any of the analyzed outcomes, thus calling into question its ergogenic potential.

As acute muscle pain occurs during resistance exercise, alleviating this exercise-induced pain through the consumption of analgesics such as paracetamol may be beneficial for performance [36]. However, isolated ingestion of paracetamol was not found to improve performance in the bench press. Comparison of our results with others in the literature is limited, given that only one similar study has been published [21]. Morgan and colleagues [21] provided 1000 mg of paracetamol 45 minutes before the participants performed knee extensions until task failure. The load for this task was initially set at 4 kg and subsequently increased by 0.5 kg every minute until task failure occurred. Time-to-task failure was similar in the placebo (396 ± 105 seconds) and paracetamol conditions (402 ± 101 seconds). While these results are valuable from a mechanistic standpoint, the protocol utilized may not necessarily apply to traditional resistance exercise protocols, where sets are generally performed with a fixed load for a given number of repetitions (e.g. 8 repetitions performed with 80% of 1RM). While we utilized a more ecologically valid protocol, we also did not find an ergogenic effect of paracetamol. Therefore, based on the currently available evidence, it does not seem that paracetamol consumption acutely enhances muscular endurance.

While paracetamol was not ergogenic, isolated caffeine ingestion increased the number of repetitions completed in the set performed to muscular failure. Compared to placebo, caffeine ingestion allowed the participants to complete approximately 1 additional repetition (*d* = 0.42). These results largely mirror those observed previously in the literature. For example, one study [37] provided 3 mg/kg of caffeine to participants before a set of bench press to muscular failure using 85% of 1RM and reported an ergogenic effect of caffeine (*d* = 0.53). A meta-analysis [38] also explored the effects of caffeine on muscular endurance and reported pooled effects similar to those observed herein (*d* = 0.42 vs. 0.38). Even though isolated caffeine elicited an ergogenic effect, caffeine + paracetamol did not enhance performance. While this may indicate that paracetamol negates the effects of caffeine, it should be considered that the data still favored the caffeine + paracetamol condition (*d* = 0.23; 95% CI: −0.10, 0.57; *p* = 0.12). Thus, the lack of an ergogenic effect might have been due to the inter-individual response variation, a topic that should be explored in future research [39].

As with the bench press, we also did not find an ergogenic effect of paracetamol on measures of isokinetic performance. Due to the paucity of data, the comparison of our results with other studies is highly limited. Using a partially similar design, Morgan and colleagues [20] provided 1000 mg of paracetamol 60 minutes before the participants performed 60 × 3 seconds maximum voluntary contractions, separated by 2 seconds of rest. Mean torque and critical torque were 3% and 4% higher in the paracetamol condition, respectively. The researchers only analyzed data across the 60 sets and did not explore set-specific effects. Based on the trajectory of decline in strength with repeated contractions, it seemed that paracetamol was effective in attenuating the decline in muscular strength starting approximately at the middle point of the session (i.e. from sets 25–30) [20]. Therefore, it might be that paracetamol ingestion is ergogenic for strength only with highly fatiguing protocols (i.e. repeated sets). These methodological differences may explain why paracetamol ingestion did not increase torque in our isokinetic protocol. Possible differences in findings between the studies also may be associated with the dose used (1500 mg vs. 1000 mg), the type of contraction (isokinetic vs. isometric), or the population studied (active vs. resistance-trained participants). An important consideration of our study is that we evaluated the effects of paracetamol as an ergogenic aid only on exercise performance, but not on outcomes related to post-exercise recovery. Roberts et al. [40] recently evaluated the effects of non-steroidal anti-inflammatory drugs (celecoxib, ibuprofen, and flurbiprofen), which have a similar pharmacology and toxicology as paracetamol. The study found that celecoxib (but not ibuprofen and flurbiprofen) attenuated the decline in MVC 4-h post plyometric exercise [40]. Future studies with a similar design may consider exploring if paracetamol may provide similar effects to celecoxib.

Consistent with previous literature, we found that caffeine was ergogenic for strength (torque), endurance (total work), and power in the isokinetic task [41–44]. These ergogenic effects are likely explained by caffeine’s ability to enhance neural drive and increase motor unit recruitment [45,46]. Effect sizes observed in this study (*d* = 0.16–0.26) are highly similar to previous studies. For example, one study provided 300 mg of caffeine and found an ergogenic effect on isokinetic outcomes ranging from *d* = 0.21 to 0.31 [41]. Similarly, a meta-analysis of 10 studies reported that caffeine ingestion increases isokinetic torque by an effect size ranging from *d* = 0.16 to 0.19 [44]. Except for total work during knee flexion at 180°/sec, we generally observed an ergogenic effect of caffeine and/or caffeine + paracetamol using slower angular velocity (i.e. 60°/sec). Indeed, previous studies also observed that caffeine ingestion produces more consistent effects at slower vs. faster angular velocities, even though this finding is not uniform in the literature [42–44]. To directly explore if there is an angular velocity-specific effect of caffeine, future studies may consider randomizing the testing order of different angular velocities.

Paracetamol consumption did not enhance performance in the Wingate test. Only two studies previously explored the effects of paracetamol on Wingate test performance. Foster et al. [22] used a protocol where 1500 mg of paracetamol was ingested 60 minutes before performing 8 bouts of the Wingate test (2-min rest). Paracetamol ingestion increased mean power by 8–9% in sprints 6–8. No significant difference was found for peak power. Using the same protocol, Delextrat et al. [23] found increases (11–14%) in mean power in bouts 2, 3, and 5, and peak power in bout 5. In accordance with our data, both studies did not find improvements in the first bout. Therefore, paracetamol consumption might be ergogenic for mean or peak power only during repeated, but not single Wingate bouts. When evaluated following the completion of the Wingate test, we found higher RPE values with paracetamol vs. placebo. Thus, our findings demonstrate that paracetamol was not ergogenic for Wingate performance, and it also increased perceived exertion during the test. In animal model studies, paracetamol consumption has been associated with a lack of motivation [47]. Hypothetically, it might be that paracetamol consumption before exercise decreases motivation, which is why the participants experienced an increase in RPE [47]. Researchers may consider exploring this hypothesis in future research. There is also evidence that paracetamol ingestion may acutely increase heart rate [18], subsequently increasing RPE. Such an effect might have occurred in our study as well, but we did not evaluate heart rate, which is something that future studies may consider.

We also did not find an ergogenic effect of caffeine in the Wingate and CMJ tests. Previous studies reported an ergogenic effect of caffeine on peak and mean power in the Wingate test [48,49]. For example, in one recent study 5 mg/kg of caffeine increased Wingate peak power, even though the effect size was small (*d* = 0.14) [48]. Previous research also explored CMJ-related outcomes (e.g. jump height) and reported an ergogenic effect of caffeine [41,42,50]. Lara and colleagues [51] found that caffeine ingestion (3 mg/kg) increased jump height by around 0.8 cm (*d* = 0.20) – an effect not observed in the present study. These divergent findings may be explained by the methodological differences between studies. Specifically, Wingate was the third test in our sequence, while the CMJ test was the fourth. This differs from several studies reporting an ergogenic effect, where the Wingate or CMJ were performed as the first (or only) test used in the protocol [41,42,48,51]. It might be the fatigue generated in the tests performed earlier in the sequence impacted the effectiveness of caffeine in the latter tests. Future studies may consider exploring whether the effects of caffeine on Wingate- and CMJ-related outcomes differ when assessed in a fatigued vs. non-fatigued state.

Caffeine ingestion has been previously associated with side effects such as anxiety, vomiting, nausea, and others [50]. However, these side effects are generally observed with higher doses (e.g. 6 mg/kg or 9 mg/kg) [50]. We used a smaller caffeine dose (3 mg/kg) and observed a low incidence of side effects. For example, only five and six participants reported experiencing side effects such as nausea, tachycardia and heart palpitations, respectively. More importantly, the incidence of side effects did not statistically differ between the conditions. Previous studies on paracetamol consumption and exercise performance did not evaluate side effects, which has been highlighted as a limitation of the literature [7]. Here, we examined the incidence of some of the most reported side effects associated with paracetamol such as rash, itching, swelling of the lips and face, lack of breath, and skin peeling [52]. As with caffeine, the incidence of side effects with paracetamol was very low and did not statistically differ between the conditions.

### 4.1. Strengths and limitations

There are several study strengths and limitations that need to be considered when interpreting the data. One of the strengths is the sample size, as we included 29 resistance-trained participants. We utilized a double-blind design and evaluated the effectiveness of the blinding. Only 7% to 28% of participants were able to identify some of the treatments received; none of the participants correctly identified all four conditions. We have also provided a comprehensive evaluation of exercise performance, thus providing a detailed insight into the effectiveness of caffeine and paracetamol. Nevertheless, there are limitations of the study that also need to be considered. One such limitation is the absolute dose of paracetamol provided to the participants. We opted for an absolute dose of 1500 mg as studies reporting an ergogenic effect used such a dose [14,15,22,23]. Additionally, we used such an approach as commercially available paracetamol is generally in absolute doses (e.g. 250 mg, 500 mg, etc.). However, this may be considered a limitation of the study, as the absolute dose of 1500 mg resulted in different relative (i.e. per kg of body mass) doses. Another limitation to consider is that we did not evaluate plasma paracetamol levels. Again, we provided paracetamol 45 min before exercise because: (a) paracetamol plasma half-life is 1.5–2.5 h [53,54]; and (b) ergogenic effects of paracetamol were previously observed with this timing of consumption [55]. It might be that we would have found different results with shorter/longer timing of consumption before exercise. The timing of paracetamol ingestion should be explored in future research.

## 5. Conclusions

We found that isolated caffeine ingestion acutely improves muscular endurance in the bench press. Isolated caffeine and/or caffeine + paracetamol ingestion was ergogenic for isokinetic muscular endurance, strength, and power, particularly at lower angular velocities. No ergogenic effects were observed for outcomes related to the Wingate and CMJ tests. Isolated paracetamol consumption did not improve performance for any of the analyzed outcomes, thus calling into question its ergogenic potential. Future research is needed to explore the influence of paracetamol dose and timing of consumption on various components of exercise performance.

## Data Availability

The datasets used in this study are available from the corresponding author upon reasonable request.

## References

[1] Del Coso J, Muñoz G, Muñoz-Guerra J. Prevalence of caffeine use in elite athletes following its removal from the world anti-doping agency list of banned substances. Appl Physiol Nutr Metab. 2011;36(4):555–18. doi: 10.1139/h11-052 21854160

[2] Grgic J, Grgic I, Pickering C, et al. Wake up and smell the coffee: caffeine supplementation and exercise performance-an umbrella review of 21 published meta-analyses. Br J Sports Med. 2020;54(11):681–688. doi: 10.1136/bjsports-2018-100278 30926628

[3] Maughan RJ, Burke LM, Dvorak J, et al. IOC consensus statement: dietary supplements and the high-performance athlete. Int J Sport Nutr Exerc Metab. 2018;28(2):104–125. doi: 10.1123/ijsnem.2018-0020 29589768

[4] Burke LM. Practical issues in evidence-based use of performance supplements: supplement interactions, repeated use and individual responses. Sports Med. 2017;47(Suppl 1):79–100. doi: 10.1007/s40279-017-0687-1 28332111 PMC5371635

[5] Jagim AR, Harty PS, Camic CL. Common ingredient profiles of multi-ingredient pre-workout supplements. Nutrients. 2019;11(2):254. doi: 10.3390/nu11020254 30678328 PMC6413194

[6] McLellan TM, Caldwell JA, Lieberman HR. A review of caffeine’s effects on cognitive, physical and occupational performance. Neurosci Biobehav Rev. 2016;71:294–312. doi: 10.1016/j.neubiorev.2016.09.001 27612937

[7] Grgic J. What is the effect of paracetamol (acetaminophen) ingestion on exercise performance? Current findings and future research directions. Sports Med. 2022;52(3):431–439. doi: 10.1007/s40279-021-01633-4 35038139

[8] Dear JW, Antoine DJ, Park BK. Where are we now with paracetamol? BMJ. 2015;351:h3705. doi: 10.1136/bmj.h3705 26163298

[9] Graham GG, Davies MJ, Day RO, et al. The modern pharmacology of paracetamol: therapeutic actions, mechanism of action, metabolism, toxicity and recent pharmacological findings. Inflammopharmacology. 2013;21(3):201–232. doi: 10.1007/s10787-013-0172-x 23719833

[10] Pickering G, Kastler A, Macian N, et al. The brain signature of paracetamol in healthy volunteers: a double-blind randomized trial. Drug Des Dev Ther. 2015;9:3853–3862. doi: 10.2147/DDDT.S81004 PMC4517518 26229445

[11] Sari DM, Rønne Pedersen J, Bloch Thorlund J, et al. Pain medication use in youth athletes: a cross-sectional study of 466 youth handball players. Transl Sports Med. 2021;4(6):914–920. doi: 10.1002/tsm2.295

[12] Tscholl PM, Vaso M, Weber A, et al. High prevalence of medication use in professional football tournaments including the world cups between 2002 and 2014: a narrative review with a focus on NSAIDs. Br J Sports Med. 2015;49(9):580–582. doi: 10.1136/bjsports-2015-094784 25878074 PMC4413681

[13] Pedersen JR, Andreucci A, Thorlund JB, et al. Prevalence, frequency, adverse events, and reasons for analgesic use in youth athletes: a systematic review and meta-analysis of 44,381 athletes. J Sci Med Sport. 2022;25(10):810–819. doi: 10.1016/j.jsams.2022.08.018 36100523

[14] Mauger AR, Jones AM, Williams CA. Influence of acetaminophen on performance during time trial cycling. J Appl Physiol. 2010;108(1):98–104. doi: 10.1152/japplphysiol.00761.2009 19910336

[15] Mauger AR, Taylor L, Harding C, et al. Acute acetaminophen (paracetamol) ingestion improves time to exhaustion during exercise in the heat. Exp Physiol. 2014;99(1):164–171. doi: 10.1113/expphysiol.2013.075275 24058189

[16] Jessen S, Eibye K, Christensen PM, et al. No additive effect of acetaminophen when co-ingested with caffeine on cycling performance in well-trained young men. J Appl Physiol. 2021;131(1):238–249. doi: 10.1152/japplphysiol.00108.2021 34013747

[17] Tomazini F, Santos-Mariano AC, Andrade-Souza VA, et al. Caffeine but not acetaminophen increases 4-km cycling time-trial performance. PharmaNutrition. 2020;12:100181. doi: 10.1016/j.phanu.2020.100181

[18] Burtscher M, Gatterer H, Philippe M, et al. Effects of a single low-dose acetaminophen on body temperature and running performance in the heat: a pilot project. Int J Physiol Pathophysiol Pharmacol. 2013;5(3):190–193. 24044039 PMC3773079

[19] Grgic J, Mikulic P. Effects of paracetamol (acetaminophen) ingestion on endurance performance: a systematic review and meta-analysis. Sports. 2021;9(9):126. doi: 10.3390/sports9090126 34564331 PMC8471630

[20] Morgan PT, Bowtell JL, Vanhatalo A, et al. Acute acetaminophen ingestion improves performance and muscle activation during maximal intermittent knee extensor exercise. Eur J Appl Physiol. 2018;118(3):595–605. doi: 10.1007/s00421-017-3794-7 29332237 PMC5805811

[21] Morgan PT, Bailey SJ, Banks RA, et al. Contralateral fatigue during severe-intensity single-leg exercise: influence of acute acetaminophen ingestion. Am J Physiol Regul Integr Comp Physiol. 2019;317(2):R346–54. doi: 10.1152/ajpregu.00084.2019 31141387 PMC6732432

[22] Foster J, Taylor L, Chrismas BC, et al. The influence of acetaminophen on repeated sprint cycling performance. Eur J Appl Physiol. 2014;114(1):41–48. doi: 10.1007/s00421-013-2746-0 24122176

[23] Delextrat A, O’Connor EM, Baker CE, et al. Acetaminophen ingestion improves repeated sprint cycling performance in females: a randomized crossover trial. Kinesiology. 2015;47(2):145–150.

[24] Derry CJ, Derry S, Moore RA. Caffeine as an analgesic adjuvant for acute pain in adults. Cochrane Database Syst Rev. 2014;12:CD009281. 25502052 10.1002/14651858.CD009281.pub3 PMC6485702

[25] Schoenfeld B, Fisher J, Grgic J, et al. Resistance training recommendations to maximize muscle hypertrophy in an athletic population: position stand of the IUSCA. Int J Strength Cond. 2021;1(1). doi: 10.47206/ijsc.v1i1.81

[26] Perrin DH. Isokinetic exercise and Assessment; Human Kinetics. Champaign (IL), USA: Human Kinetics; 1993.

[27] Smith JC, Hill DW. Contribution of energy systems during a Wingate power test. Br J Sports Med. 1991;25(4):196–199. doi: 10.1136/bjsm.25.4.196 1839780 PMC1479034

[28] Bertucci WM, Hourde C. Laboratory testing and field performance in BMX riders. J Sports Sci Med. 2011;10(2):417–419. 24137057 PMC3761846

[29] Hofman N, Orie J, Hoozemans MJM, et al. Wingate test as a strong predictor of 1500-m performance in elite speed skaters. Int J Sports Physiol Perform. 2017;12(10):1288–1292. doi: 10.1123/ijspp.2016-0427 28253027

[30] Watson RC, Sargeant TL. Laboratory and on-ice test comparisons of anaerobic power of ice hockey players. Can J Appl Sport Sci. 1986;11(4):218–224. 3815713

[31] Zouhal H, Saeidi A, Salhi A, et al. Exercise training and fasting: current insights. Open Access J Sports Med. 2020;11:1–28. doi:10.2147/OAJSM.S224919 32021500 PMC6983467

[32] Frikha M, Chaâri N, Mezghanni N, et al. Influence of warm-up duration and recovery interval prior to exercise on anaerobic performance. Biol Sport. 2016;33(4):361–366. doi: 10.5604/20831862.1221830 28090140 PMC5143772

[33] Borg G. Perceived exertion as an indicator of somatic stress. Scand J Rehabil Med. 1970;2(2):92–98. doi: 10.2340/1650197719702239298 5523831

[34] Cook DB, O’Connor PJ, Oliver SE, et al. Sex differences in naturally occurring leg muscle pain and exertion during maximal cycle ergometry. Int J Neurosci. 1998;95(3–4):183–202. doi: 10.3109/00207459809003340 9777439

[35] Saunders B, de Oliveira LF, da Silva RP, et al. Placebo in sports nutrition: a proof-of-principle study involving caffeine supplementation. Scand J Med Sci Sports. 2017;27(11):1240–1247. doi: 10.1111/sms.12793 27882605

[36] Lixandrão ME, Roschel H, Ugrinowitsch C, et al. Blood-flow restriction resistance exercise promotes lower pain and ratings of perceived exertion compared with either high- or low-intensity resistance exercise performed to muscular failure. J Sport Rehabil. 2019;28(7):706–710. doi: 10.1123/jsr.2018-0030 30040033

[37] Grgic J, Pickering C, Bishop DJ, et al. CYP1A2 genotype and acute effects of caffeine on resistance exercise, jumping, and sprinting performance. J Int Soc Sports Nutr. 2020;17(1):21. doi: 10.1186/s12970-020-00349-6 32295624 PMC7161272

[38] Polito MD, Souza DA, Casonatto J, et al. Acute effect of caffeine consumption on isotonic muscular strength and endurance: a systematic review and meta-analysis. Sci Sport. 2016;31(3):119–128. doi: 10.1016/j.scispo.2016.01.006

[39] Pickering C, Kiely J. Are the current guidelines on caffeine use in sport optimal for everyone? Inter-individual variation in caffeine ergogenicity, and a move towards personalised sports nutrition. Sports Med. 2018;48(1):7–16. doi: 10.1007/s40279-017-0776-1 28853006 PMC5752738

[40] Roberts BM, Sczuroski CE, Caldwell AR, et al. NSAIDs do not prevent exercise-induced performance deficits or alleviate muscle soreness: a placebo-controlled randomized, double-blinded, cross-over study. J Sci Med Sport. 2024;27(5):287–292. doi: 10.1016/j.jsams.2024.02.002 38383211

[41] Venier S, Grgic J, Mikulic P. Acute enhancement of jump performance, muscle strength, and power in resistance-trained men after consumption of caffeinated chewing gum. Int J Sports Physiol Perform. 2019;14(10):1415–1421. doi: 10.1123/ijspp.2019-0098 30958062

[42] Venier S, Grgic J, Mikulic P. Caffeinated gel ingestion enhances jump performance, muscle strength, and power in trained men. Nutrients. 2019;11(4):937. doi: 10.3390/nu11040937 31027246 PMC6520843

[43] Duncan MJ, Thake CD, Downs PJ. Effect of caffeine ingestion on torque and muscle activity during resistance exercise in men. Muscle And Nerve. 2014;50(4):523–527. doi: 10.1002/mus.24179 24435882

[44] Grgic J, Pickering C. The effects of caffeine ingestion on isokinetic muscular strength: a meta-analysis. J Sci Med Sport. 2019;22(3):353–360. doi: 10.1016/j.jsams.2018.08.016 30217692

[45] Bowtell JL, Mohr M, Fulford J, et al. Improved exercise tolerance with caffeine is associated with modulation of both peripheral and central neural processes in human participants. Front Nutr. 2018;5:6. doi: 10.3389/fnut.2018.00006 29484298 PMC5816050

[46] Black CD, Waddell DE, Gonglach AR. Caffeine’s ergogenic effects on cycling: neuromuscular and perceptual factors. Med Sci Sports Exerc. 2015;47(6):1145–1158. doi: 10.1249/MSS.0000000000000513 25211364

[47] Chen Z, Wei H, Pertovaara A, et al. Anxiety- and activity-related effects of paracetamol on healthy and neuropathic rats. Pharmacol Res Perspect. 2018;6(1):e00367. doi: 10.1002/prp2.367 29417759 PMC5817821

[48] Marinho AH, Silva-Cavalcante MD, Cristina-Souza G, et al. Caffeine, but not creatine, improves anaerobic power without altering anaerobic capacity in healthy men during a Wingate anaerobic test. Int J Sport Nutr Exerc Metab. 2024;34(3):137–144. doi: 10.1123/ijsnem.2023-0191 38458183

[49] Grgic J. Caffeine ingestion enhances Wingate performance: a meta-analysis. Eur J Sport Sci. 2018;18(2):219–225. doi: 10.1080/17461391.2017.1394371 29087785

[50] Lara B, Gonzalez-Millán C, Salinero JJ, et al. Caffeine-containing energy drink improves physical performance in female soccer players. Amino Acids. 2014;46(5):1385–1392. doi: 10.1007/s00726-014-1709-z 24615239

[51] de Souza Jg, Del Coso J, Fonseca FDS, et al. Risk or benefit? Side effects of caffeine supplementation in sport: a systematic review. Eur J Nutr. 2022;61(8):3823–3834. doi: 10.1007/s00394-022-02874-3 35380245

[52] Popiołek I, Piotrowicz-Wójcik K, Porebski G. Hypersensitivity reactions in serious adverse events reported for paracetamol in the eudravigilance database, 2007-2018. Pharmacy. 2019;7(1):12. doi: 10.3390/pharmacy7010012 30658389 PMC6473647

[53] Forrest JA, Clements JA, Prescott LF. Clinical pharmacokinetics of paracetamol. Clin Pharmacokinet. 1982;7(2):93–107. doi: 10.2165/00003088-198207020-00001 7039926

[54] Prescott LF. Kinetics and metabolism of paracetamol and phenacetin. Br J Clin Pharmacol. 1980;10(Suppl 2):291S–2918. doi: 10.1111/j.1365-2125.1980.tb01812.x 7002186 PMC1430174

[55] Pagotto FD, Paradisis G, Maridaki M, et al. Effect of acute acetaminophen injestion on running endurance performance. J Exerc Physiol Online. 2018;21(3):106–118.

